# Prevalence, Patterns and Self-Perceived Effects of Pornography Consumption in Polish University Students: A Cross-Sectional Study

**DOI:** 10.3390/ijerph16101861

**Published:** 2019-05-27

**Authors:** Aleksandra Diana Dwulit, Piotr Rzymski

**Affiliations:** Department of Environmental Medicine, Poznan University of Medical Sciences, 60-806 Poznan, Poland; ad.dwulit@gmail.com

**Keywords:** pornography, cross-sectional study, university students, self-perceived effects, questionnaire survey

## Abstract

This cross-sectional online survey of Polish students (*n* = 6463) assessed the frequency and patterns of pornography consumption, its self-perceived effects, the prevalence of self-perceived pornography addiction, and opinions on the potential effects of pornography and its legal status. Nearly 80% of students have been exposed to pornography (median age of first exposure: 14 years). Streaming videos were decidedly the most frequent form of use. In the subset of current users (*n* = 4260), daily use and self-perceived addiction was reported by 10.7% and 15.5%, respectively. The majority of those surveyed did not report any negative effects of pornography use on their sexual function, sexual, and relationship satisfaction. Instead, over one-quarter of students in relationships reported beneficial effects on its quality. The most common self-perceived adverse effects of pornography use included: the need for longer stimulation (12.0%) and more sexual stimuli (17.6%) to reach orgasm, and a decrease in sexual satisfaction (24.5%). Females and males with a Body Mass Index >25 kg/m^2^ more often reported a self-perceived decrease in relationship quality associated with pornography use. Age of first exposure was significantly associated with reported need for longer stimulation and more sexual stimuli to reach orgasm when using pornography, decrease in sexual satisfaction, and quality of romantic relationship, neglect of basic needs and duties due to pornography use, and self-perceived addiction in both females and males. The highest odds ratios were always observed for age <12 years in reference to exposure at >16 years. In the opinion of most of the surveyed students, pornography may have adverse effects on human health, although access restrictions should not be implemented. The study gives a broad insight into the pornography consumption in young Polish adults.

## 1. Introduction

The online pornography industry has been developing at a fast pace due to a global increase of Internet accessibility and technological progress, particularly in streaming media that allow users to continuously watch content, usually a video, without the need to download it [1]. It is, thus, no surprise that explicit material is now ubiquitously and readily available on the Internet while intended and unintended exposure to it may sometimes be difficult to avoid [2,3]. 

According to statistics shared by Pornhub, a major online website with explicit content, the group of pornography consumers is steadily increasing and it is mostly represented by men (over 70% of all users) and young adults, below 34 years old [4]. In line with this data, over 70% of adult US citizens, aged 18–30 years old, admit to watching online pornography at least once a month while nearly 60% of college students admitted to its consumption once a week [5]. Adolescents also constitute an important group of intentional online viewers of pornography with user rates in countries such as Taiwan and Sweden estimated at level up to 59% and 96%, respectively [6,7]. 

Although pornography has a long history, the new technologies have undoubtedly led it to new heights. It is now offered in almost unlimited sexual diversity via free-of-charge online websites accessible through any device with Internet access, mostly in the form of video pornography, which was reported to be the most sexually arousing of all forms of explicit material [8,9]. The ease, diversity, and arousal strength with which online pornography can reach its consumers indicates that it may operate as a supernormal stimulus [10,11,12]. There are, however, controversies over the exact effects that it may potentially exert on its consumers. Some studies reported that long-term use correlates with erectile dysfunction, decreased libido [12,13,14,15], higher interest in pornography than sexual contacts with real partners [13,16], and lower sexual and relationship satisfaction [15,17,18,19,20]. One should, however, note that the majority of these investigations cannot assess the causality and, furthermore, that there are some other research that clearly produced the contrary observations. For example, some cross-sectional studies and experimental investigations failed to find an association between erectile dysfunction and pornography use [21,22,23,24], some research also suggest that men with sexual dysfunctions, such as erectile dysfunction, may tend to use more pornography, including patterns they self-perceive as problematic [24]. There are also investigations reporting a positive correlation between pornography use in men and their sexual arousal, desire for solo and partnered sexual behaviors [23], as well as studies suggesting that pornography use may reduce risky sexual behaviors [25], indicating that women involved in long-term relationships that use pornography more frequently may reveal increased sexual desire towards their partners and report higher desire for sexual variety [26], and highlighting that shared viewing of pornography in heterosexual couples correlates with increase of sexual satisfaction [27]. All in all, there is a need to further explore pornography use among different groups and by using various research approaches encompassing cross-sectional, case-control, and prospective cohort studies. 

The pornography addiction is not a formally recognized disorder in the ICD-10 or DSM-5 classifications, therefore, some investigators have referred to it as “self-perceived pornography addiction” [28,29,30]. The evidence from neurobiological studies indicates that it may fit into the general addiction framework, and share similar mechanisms with those observed in addictions to chemical substances [31,32,33,34,35] although controversies in this regard exist [35,36] and some alternative models based on compulsivity, impulsivity or moral incongruence were suggested to describe high and problematic consumption of pornography [24,36]. Some preliminary case reports suggest that naltrexone, predominantly used in the treatment of alcohol and opioid dependence, can be successfully applied in patients with compulsive pornography use [37,38]. 

It is known that most individuals actively explore sexual behaviors and gain sexual experience until their mid-20s [39,40]. It can, therefore, be hypothesized that for young adults the consumption of pornography may represent some sort of substitute for these activities or be a part of them. This, in turn, creates a need to understand how these individuals may perceive pornography. For this reason, some studies have addressed this issue by investigating groups of college students but sample size was often not high or limited to only one gender [41,42,43,44,45,46]. This is, therefore, of interest to conduct further studies that would survey large groups and assess to which extent individual characteristics may affect differentiate the patterns of pornography use. For example, it would be interesting whether particular personality traits can be associated with pornography use as some previous studies have reported that they may influence sexual activities, such as novelty seeking [47]. The sexual activities might also be influenced by physical characteristics such as Body Mass Index [48], yet not much is known whether it may be associated in any way with pornography use. Moreover, patterns of pornography consumption may depend on whether studied individuals are single or in a relationship; it was reported recently that the latter group tend to use it less often [49]. 

The aim of the present cross-sectional online survey study was to assess the prevalence of pornography use, age of first exposure, patterns of pornography consumption, attempts to cease its use and self-reported effects of such cessation, self-perceived effects of pornography use, and prevalence of self-perceived addiction to pornography among Polish female and male university students aged 18–26 years old. The associations of these parameters with body mass index (BMI), status of romantic relationship, and eighteen self-reported personality traits were evaluated. Moreover, students opinions on the effects associated with pornography use and its legal status were also assessed. The study provides a broad insight into various aspects of pornography use in young adults.

## 2. Materials and Methods

### 2.1. Survey

To explore the patterns of pornography consumption, self-perceived effects of its use, and how it is generally perceived by Polish university students, an anonymous, online survey based on a self-designed, structured questionnaire was conducted. As previously indicated, research based on online questionnaire creates the opportunity to collect data nationwide and to reach specific groups of individuals [50,51]. Anonymity of the study was assured to eliminate the effect of embarrassment that may be associated with pornography consumption [52]. As recently evidenced, surveying adolescent pornography consumption does not increase its subsequent use by study participants [53,54].

The questionnaire employed in the present research aimed to assess:

1. the prevalence of pornography use and age of first exposure in the studied group;
2. the patterns of pornography exposure: (i) the forms and frequency of use, (ii) an average length of single use, (iii) use of private (incognito mode) and multi-window browsing when watching online pornography, and (iv) consumption outside a place of residence;
3. the frequency of attempts to cease pornography use in the group of its current users, and prevalence and severity of associated effects (using a four-point scale);
4. the self-perceived effects of pornography regarding (i) changes in the type of consumed content such as switching to a novel genre, progression to more extreme (violent) material, viewing content not matching sexual orientation, (ii) sexual satisfaction, (iii) romantic relationship quality, (iv) changes in time of stimulation and number of stimuli needed to reach an orgasm when using pornography, and (v) neglecting basic needs (e.g., sleeping, eating) and duties (e.g., home-associated, occupational) due to pornography use;
5. the prevalence of self-perceived addiction to use of pornography; and
6. general opinion on pornography: (i) the effect it may have on social relationships, mental health and sexual performance, and psychosocial development in childhood and adolescence, (ii) the possibility that it may cause addiction, (iii) border age of harmless pornography exposure, and (iv) the current legal status of pornography in Poland (an open access to adult pornography).

Inclusion criteria for the study were Polish nationality, age 18–26 years old, female or male gender, and university student. These criteria were verified by answers given to the corresponding survey questions. Only completed questionnaires were analyzed. The demographic characteristics of each surveyed individual included gender, studied field of science (medical, biological, social, or other), and BMI (calculated from reported weight and height). The questionnaire was made available online for the period of a year (February 2017–August 2018). Since it was previously reported that personal traits may influence sexual activities such as novelty seeking [47], selected personality traits were self-identified by students being able to choose one the two opposite characteristics that suited them best. The following pairs of traits based on the list given by DeNeve and Cooper [55] were prepared; they included: introversive/extroversive, optimistic/pessimistic, confident/shy, curious/uninquisitive, sensitive/insensitive, happy/sad, calm/aggressive, trustful/conscious, and social/antisocial.

In order to approach the largest possible group of students, invitations to complete the questionnaire were sent by universities, and were made available through social media and web portals. The study was approved by the Local Bioethical Committee of the Poznan University of Medical Sciences, Poznan, Poland (approval #68/17 issued 5th January, 2017). 

### 2.2. Statistical Analyses

A total of 9070 questionnaires were collected of which 2606 did not met inclusion criteria (30.5%), or were incomplete (69.5%) and were, therefore, excluded to avoid fallacious results. The analyses were performed using Statistica v.13.1 (StatSoft Inc., Tulsa, OK, USA). Since most of the data did not meet the assumption of Gaussian distribution (Shapiro–Wilk test; *p* < 0.05), nonparametric methods were used to test the results. To evaluate differences between two and three independent groups, the Mann–Whitney U test and Kruskal–Wallis ANOVA were used, respectively. Differences in dichotomous data were assessed by the Pearson’s χ2 test. To evaluate the associations between age of first exposure, demographical variables and personal traits, and self-reported effects of pornography use (need for longer stimulation and need for more sexual stimuli to reach orgasm when using pornography, decrease in sexual satisfaction, decrease in romantic relationship quality, neglecting basic needs, neglecting duties and self-perceived pornography addiction), as well as the occurrence of adverse effects of pornography cessation, the classical odds ratios (ORs) with a 95% confidence interval (95%CI) were calculated according to the formulas given by Bland and Altman [56] using MedCalc (MedCalc, Ostend, Belgium). The age of first exposure to pornography was classified into four categories based on the quartile distribution in the study population: ≤12, 13–14, 15–16, and ≥16 years. A *p*-value of *p* < 0.05 was considered as statistically significant. 

## 3. Results

### 3.1. Demographic Characteristics

The studied group constituted of 6463 students (2633 male and 3830 female), aged 18–26 years old, representing medical (14.4%), biological (7.3%), social (19.2%), and other (59.1%) sciences. The demographic characteristics of the analyzed population are presented in Table 1. 

### 3.2. The Prevalence of Pornography Use

The exposure to pornography was declared by 78.6% subjects (*n* = 5083; 3004 female and 2079 male). In this subset, the current users constituted 83.8% (*n* = 4260; 2520 female and 1740 male), while the rest (*n* = 823; 484 female and 339 male) reported to successfully cease its use. The prevalence of exposure was similar in female (78.4%) and male students (79.0%) (*p* > 0.05, χ2 test). The mean ± SD age of first pornography exposure was 14.1 ± 3.0 (median 14.0) with no difference between males and females (*p* > 0.05, Mann–Whitney U test). No association between this age and any personality trait, relationship status was identified (*p* > 0.05 in all cases, χ2 test).

Compared to students never exposed to pornography (*n* = 1380), female and male, consuming pornography did not differ in BMI (*p* > 0.05, Kruskal–Wallis ANOVA). However, the frequency of pornography use was higher among females engaged in a romantic relationship if compared to singles (64.0 vs. 60%; *p* < 0.05, χ2 test). The percentages of women perceiving themselves as social and men perceiving themselves as trusting were higher in the group exposed to pornography (73.6 vs. 70.2% and 58.9 vs. 53.8%, respectively; *p* < 0.05 in both cases, χ2 test). No other associations between prevalence of pornography consumption and personality traits in female and male students were identified (*p* > 0.05 in all cases, χ2 test).

### 3.3. Patterns of Pornography Use

**Figure 1:**
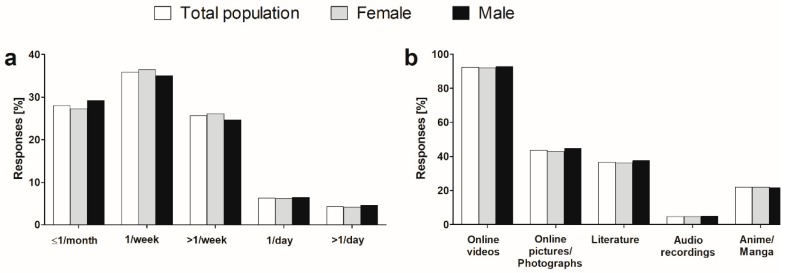
Frequency of pornography consumption (**a**) and its forms (**b**) in the surveyed group of studied university students (*n* = 4260).

Within the subset of current consumers (*n* = 4260), the most often reported frequency of use of explicit material was once per week. Daily use was reported by 10.7%, with no difference between female and male students (*p* > 0.05, χ2 test) (Figure 1a). The daily users, both female and male students, did not differ from those using pornography less frequently in terms of BMI (*p* > 0.05, Mann–Whitney U test) as well as status of romantic relationship and any personality trait (*p* > 0.05 in all cases, χ2 test). 

Online videos were decidedly the most often used form in the total surveyed population and within both sex subsets. Other forms included photography, literature, anime/manga, and very sporadically, audio recordings (Figure 1b). The estimated average length of single pornography use did not exceed 1h in case of 86.8% of surveyed. The majority of students admitted to use of private mode (76.5%, *n* = 3256) and multiple windows (51.5%, *n* = 2190) when browsing online pornography. Use of place outside residence was declared by 33.0% (*n* = 1404). None of these patterns differed between female and male students (*p* > 0.05 in all cases, χ2 test).

### 3.4. Attempts to Cease Pornography Use

Among those surveyed who declared themselves to be current pornography consumers (*n* = 4260), 51.0% admitted to making at least one attempt to give up using it with no difference in the frequency of these attempts between males and females (*p* > 0.05; χ2 test). 72.2% of those attempting to quit pornography use indicated the experience of at least one associated effect, and the most frequently observed included erotic dreams (53.5%), irritability (26.4%), attention disturbance (26.0%), and sense of loneliness (22.2%) (Table 2). Compared to males engaged in romantic relationships, singles reported a higher incidence of the occurrence of adverse effects during pornography cessation—OR (95%CI) was 1.22 (1.01–1.5) (*p* < 0.05). BMI and personal traits were not found to significantly differentiate the incidence of cessation-associated effects within subsets of female and male students.

### 3.5. Self-Perceived Effects of Pornography Use

**Figure 2:**
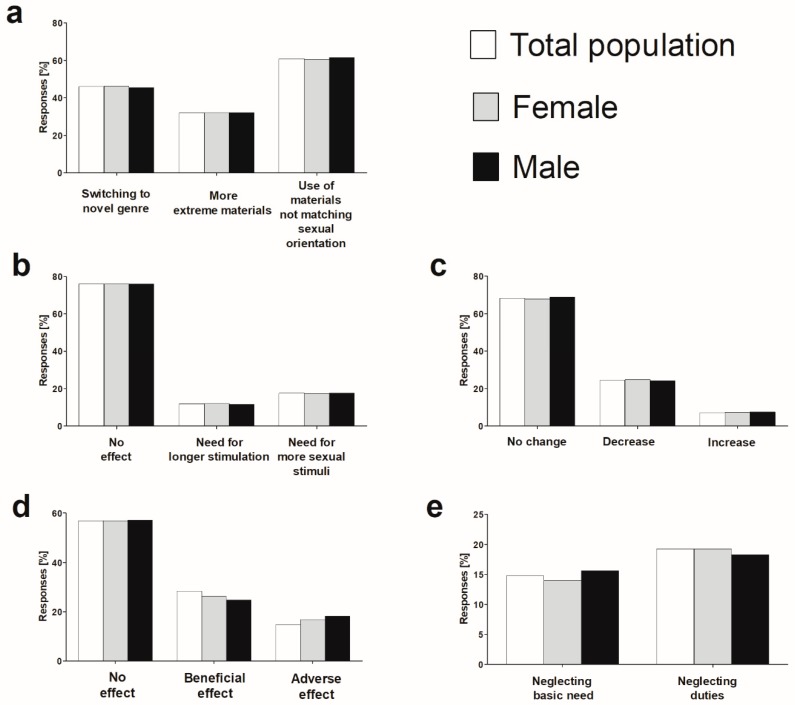
The self-perceived changes in the pattern of pornography use in the course of its consumption (**a**), the reported self-perceived effects of pornography consumption on sexual performance during its consumption (**b**), sexual satisfaction (**c**), romantic relationship quality (**d**), and neglecting basic needs and duties at least once in their lifetime (**e**) in the surveyed group of university students (*n* = 4260).

The rate of students currently using pornography (*n* = 4260) that feel embarrassed about this activity amounted to 49.1%, and was significantly higher in females than males (57.8 vs. 42%; *p* < 0.05, χ2 test). Various changes of pattern of pornography use occurring in the course of the exposure period were reported: switching to a novel genre of explicit material (46.0%), use of materials that do not match sexual orientation (60.9%) and need to use more extreme (violent) material (32.0%) (Figure 2a). The latter was more frequently reported by females considering themselves as curious compared to those regarding themselves as uninquisitive (32.3 vs. 26.7%; *p* < 0.01, χ2 test), and aggressive males compared to calm (32.8 vs. 24.2%; *p* < 0.05 χ2 test). Longer stimulation and more sexual stimuli needed to reach an orgasm when using pornography was reported by 12.0% and 17.6%, of those surveyed, respectively (Figure 2b). Most of the studied subjects did not perceive any negative effects of pornography use on sexual satisfaction and relationship quality with respectively 7% and 28% reporting a beneficial impact on these parameters (Figure 2c,d).

The odds for a decrease in relationship quality were higher in female and male students with BMI ≥ 25 (OR = 1.44, 95%CI: 1.09–1.92, *p* < 0.01, and OR = 1.35, 95%CI: 1.02–1.79, *p* < 0.05, respectively) in reference to sex subsets of BMI < 25 kg/m^2^, as well as in females and males who reported embarrassment about their pornography use (OR = 3.38; 95%CI: 2.65–4.31, *p* < 0.001 and OR = 4.68, 95%CI: 3.45–6.36, *p* < 0.001) in reference to their counterparts not experiencing it. The neglect of basic needs (e.g., food or sleep) and duties (e.g., at home, work) because of pornography use was experienced at least once by 14.8 and 19.3% of students, respectively (Figure 2e). The odds for longer stimulation and more sexual stimuli needed to reach orgasm with pornography, decrease in sexual satisfaction and quality of romantic relationship, and neglect of basic needs and duties were higher in females and males exposed earlier to pornography with the highest OR (95%CI) values always observed for individuals exposed at <12 years compared to those exposed at >16 years (Table 3). No other significant associations between reported effects of pornography use and demographic parameters or personality traits of female and male students were identified. 

### 3.6. Self-Perceived Pornography Addiction

The prevalence of self-perceived addiction to pornography in the total studied population (*n* = 6463) was 12.2%, while in the subset of current users (*n* = 4260) it amounted to 15.5% (*n* = 787) with no difference observed between female and male students (*p* > 0.05, χ2 test). The association between age of first exposure to pornography and addiction is shown in Table 3. For females and males the ORs (95%CI) were 4.23 (2.85–6.28) and 7.25 (4.16–12.63), respectively, for the lowest quartile of initial exposure age in comparison with the highest quartile. 

The BMI and romantic relationship were not associated with self-perceived addiction in male and female students (*p* > 0.05 in all cases, χ2 test). Moreover, no characteristic personality trait was revealed by individuals with self-perceived addiction to pornography except in the male subset in which a higher frequency of introverted persons was found when compared to male students not declaring an addiction (71.3% vs. 64.5%; *p* < 0.05, χ2 test). The OR (95%CI) for self-perceived addiction in introversive males was 1.31 (1.01–1.71, *p* < 0.05) in reference to the group of extroversive individuals. 

### 3.7. General Opinion on Pornography

In the opinion of the majority of the surveyed students, pornography use may have a negative effect on the quality of social relationships (58.7%), mental health (63.9%) and sexual performance (67.7%), as well as negatively affect psychosocial development in childhood and adolescence (78.1%). These opinions were not diversified between sex subsets (*p* > 0.05, χ2 test) except for the latter which was expressed more frequently by female students (82.3 vs. 72.1%; *p* < 0.001, χ2 test). Compared to their male counterparts, females more often indicated that there is a safe age for pornography exposure (37.6 vs. 31.7%; *p* < 0.001, χ2 test), estimated in both sex subsets at mean ± SD (median) of 17 ± 2 (17) years (*p* > 0.05, Mann–Whitney U test). Most of the surveyed students agreed as to the existence of pornography addiction on a minor (26.8%, *n* = 1732) or wide scale (66.6%, *n* = 4306). In view of 67.8% (*n* = 4381) the current law in Poland (open access to adult materials) regarding pornography should not be subject to any modification, 24.1% (*n* = 1558) advocated for access restrictions, and the rest had no point of view in this regard. Opinions on both matters did not differ between female and male students (*p* > 0.05 in both cases, χ2 test). 

## 4. Discussion

There has been a continuous interest in the study of various aspects of pornography consumption. The present study explores these issues in university students aged 18–26 years old—a group which can be expected to be sexually active. As shown, in the United States the average age of first sexual intercourse is 17–18 years [57]. The results of the present study offer an insight into the prevalence and patterns of pornography use, and the way it is perceived in the group of university students in Poland. It demonstrates that the majority of students use pornography and that unsurprisingly, online streaming videos are the most popular form of use, as they can currently be easily reached with any device with Internet access. 

The rate of exposure as reported here falls within ranges observed for young adults in previous studies [58,59]. According to statistical data provided annually by the largest online pornography service Pornhub and observations from various epidemiological studies, the prevalence of use, particularly on a regular basis, is higher among males [4,45,60,61]. Similar conclusions were formulated in previous research employing a smaller sample size of Polish (*n* = 1135) and German (*n* = 1303) students [59]. Contrary to this, the present investigation found no significant difference between sex subsets, not only in the prevalence of pornography consumption, but also its frequency. However, recent analysis has shown that the incidence of women using pornography in various world regions is increasing [62] and, subsequently, more of them may be willing to admit it. It is also possible that the high prevalence of pornography use in females as observed in the present study is, to some extent, a result of volunteer bias—an anonymous online survey may attract consumers of pornography more than individuals not associated with explicit materials. 

A number of previous studies have focused on potential negative outcomes of pornography [63]. The present research demonstrated that nearly 25% and 15% of surveyed students perceived that pornography use adversely affects their sexual and relationship satisfaction, respectively. However, it is worth noting that majority of the surveyed individuals did not report any negative effect on their sexual satisfaction, and did not note any changes to sexual performance that would occur over the course of pornography consumption. Moreover, majority of those in relationship did not perceive that pornography had some negative effect on the quality of relationship, and over one-quarter actually indicated that pornography had beneficial effect on it. Interestingly, despite that majority of students did not note any negative effects on their own sexual function, sexual and relationship satisfaction, they mostly expressed an opinion that pornography use may adversely affect human health. This, in turn, may potentially indicate that how the pornography is perceived by young adults may not be driven by their own experience but by cultural factors, and opinions formulated by authorities and media.

However, a design of the present study cannot conclude on causality, the discrepancies in self-perceived effects of pornography use (negative, positive, or none) suggest its outcomes may potentially be associated with individual characteristics. Apart from BMI and age of first exposure, which are discussed later, the variables, such as baseline sexual satisfaction, history of sexual activities (number of partners, age of first sexual intercourse etc.), compulsivity, and impulsivity may also be important to consider. Further studies are required to explore these issues although they may be difficult to establish on basis of cross-sectional studies. Given the fact the high frequency of pornography consumption among young adults, it can be hypothesized that the context of its use may be crucial in understanding the potentially associated effects. For example, a number of studies have shown that individual pornography use may be negatively correlated with partner’s sexual satisfaction [64] while a recent investigation indicated that shared use may actually be positively associated with promotion of sexual interaction between partners and their sexual satisfaction [65]. It is also plausible that individuals with some sexual dysfunction may tend to use more pornography, highlighting a need for longitudinal studies in which baseline sexual characteristics of enrolled subjects are established. 

As demonstrated in the present study, women using pornography more often reported disgust, guilt, and embarrassment [27], and this may also limit their willingness to report or discuss any association with explicit material [66]. This advocates the use of anonymous online surveys in epidemiological studies on pornography exposure although they also introduce number of limitations as discussed later. The present study highlights that over half of female students using pornography, a significantly higher number than in the case of their male counterparts, report to be embarrassed by this activity. This difference may arise from cultural influences and much lower acceptance of pornography consumption among women compared to men, and that some women may more often associate it with an act of infidelity, although contradictory findings were reported in this respect [67,68,69]. It could be hypothesized that such embarrassment could induce distress associated with pornography use. The present study also found that women who are embarrassed about their pornography activity more often perceive that its consumption negatively impact a romantic relationship quality. However, one should also note that such an association was even more frequent in male students. Feeling ashamed of pornography consumption may impede discussing it with a partner, and potentially undermine trust in a relationship. Altogether, it supports the notion that partners should openly discuss the pornography use with each other. 

With some exceptions, none of personality traits, which were self-reported in this study, differentiated the studied parameters of pornography. These findings support the notion that access and exposure to pornography are presently issues too broad to specify any particular psychosocial characteristics of its users. However, an interesting observation was made regarding consumers who reported a need to view increasingly extreme pornographic content. As shown, frequent use of explicit material may potentially be associated with desensitization leading to a need to view more extreme content to reach similar sexual arousal [32]. Nevertheless, it was recently evidenced that the pornography industry does not produce increasingly more material presenting violent and degrading acts, and that streaming videos presenting such acts receive less views and lower rankings from online viewers [70]. As indicated by Italian research surveying high school students, a minority of them (18.8%) were exposed to violent/degrading material with a lower rate observed for females [71]. The present study found that a need to use more extreme pornography material was more frequently reported by males describing themselves as aggressive. A link between pornography and aggression has been studied previously: intentional exposure to violent material over time predicted an almost six-fold increase in the odds of self-reported sexually aggressive behavior [72]. One should, however, note the present findings cannot exclude the possibility of reverse causation (aggressive males more frequently preferring violent pornographic material). This would require case-controlled or cohort studies. Interestingly, in the present study females reporting a gradual need to explore more violent explicit materials more frequently perceived themselves as curious. It could, therefore, be hypothesized that violent pornography may attract females with a specific interest in sexual exploration, although this would also require further investigation. 

An interesting observation of this research is that overweight/obese (≥25 kg/m^2^) female and male students perceived that pornography has negative effect on relationship quality more often than those with BMI <25 kg/m^2^. For young adults, pornography may model their sexual perception and expectations, and has been reported to serve as a source of sexual information [3]). This may not unambiguously lead to adverse effects if pornography is not regarded as a primary source of such information and, as a fantasy, it is not mistaken for sexual reality [73]. Pornographic materials often present unnatural or even extreme acts by actresses and actors who, in order to adapt to the promoted type of physical appearance, often undergo plastic surgeries or use pharmaceuticals to sustain the state of erection [74]. Altogether, this may lead to the generation of sexual demands which are often impossible to meet, and discrepancies between physical attractiveness as presented in pornography and that of the exposed subjects, particularly those who are overweight or obese. It can be hypothesized that pornography may further magnify the effect, already observed in previous studies, in which heavier women were judged by their male partners as lower in attractiveness/vitality and as poorer matches to their partners’ ideals of attractiveness [75]. Moreover, high BMI has been shown to be a significant predictor of erectile dysfunction [76], an association which could also lead to a potential discrepancy between male sexual skills and those presented by pornography actors. 

Age of first exposure to explicit material was associated with increased likelihood of negative effects of pornography in young adults—the highest odds were found for females and males exposed at 12 years or below. Although a cross-sectional study does not allow an assessment of causation, this finding may indeed indicate that childhood association with pornographic content may have long-term outcomes. It has been previously suggested that early exposures support adherence to unhealthy notions of sexual relations [77]. The present study shows that individuals exposed earlier were more likely to report neglecting basic needs and duties at least once in their lifetime due to pornography use, and had perceived its negative effects on relationships quality more frequently. Whether these effects result from hypofrontal syndromes manifested by compulsivity, emotional lability, and impaired judgment is unknown, although such effects have already been reported among pornography consumers [32,34]. The present study also suggests that earlier exposure may be associated with potential desensitization to sexual stimuli as indicated by a need for longer stimulation and more sexual stimuli required to reach orgasm when consuming explicit material, and overall decrease in sexual satisfaction. As shown in neuroimaging study, exposure to pornography may lead to down-regulation of the reward system in adults via a decreased volume of caudate gray matter and its altered functional connectivity with prefrontal cortex [32]. Finally, earlier exposed female and male students had higher odds for self-perceived addiction to pornography—a phenomenon observed at a rather higher rate of 12.2% in the surveyed group. One should note, however, that this rate does not reflect whether the studied subjects were addicted to pornography in a neurobiological sense. As recently indicated, self-perceived addiction may not always be an accurate indicator of problematic pornography use [29]. Nevertheless, previous investigations have already shown that if such a perception is present it is more often associated with increased psychological distress [78]. Overall, as exposure to online pornographic content is almost unavoidable for young generations, the findings of the present study support the notion that protection of children from too early exposure should be prioritized. 

Restriction to pornography access has become a subject of political discussion. The majority of countries, including Poland, allow unrestricted access to adult pornography by adult individuals. Recently, in response to an increase in the rate of documented rapes, the government of Nepal banned pornography distribution. In the present study, the surveyed students often indicated that pornography exposure may have an adverse outcome on social relationships, mental health, sexual performance, and may affect psychosocial development in childhood and adolescence. Despite this, the majority of them did not support any need for restrictions to pornography access. In the long-term, a law that limits access to pornography, bans specific pornography websites, or implements age-verification systems may be difficult or expensive to fully enforce. However, countries such as the United Kingdom are considering restrictions based on age-verification systems for online pornography to protect children from exposure, and ensure that only adults (≥18 years old) will have access to explicit content. 

Although the present research reveals some valuable information regarding pornography consumption within a relatively large and homogeneous group of university students, there are a number of limitations which need to be outlined for cautious data interpretation. Firstly, the multiple comparisons performed when analyzing data without correction increase the probability of type 1 errors. However, a simple Bonferroni correction could potentially be overly conservative, and increase the risk of type II errors [79]. Nevertheless, the lack of such correction must be taken into account in the interpretation of the findings of the study. Moreover, the anonymous and online character of the survey excluded the possibility of verifying the data. Additionally, the reported effects of pornography use were self-perceived by surveyed individuals, and were not confirmed on clinical level. Importantly, a cross-sectional study design does not allow any identification of causation. This also relates to classical ORs calculated for associations between age of first exposure to pornography and self-perceived effects of pornography use. The age of first exposure reported by surveyed students should be also treated with caution rather as a rough estimate. As already outlined, volunteer bias could not be fully excluded under the anonymous study design and must be taken into account when interpreting the high prevalence of pornography use, particularly in females, or the high rate of self-perceived addiction. Moreover, it is unknown to what extent pornography use in the studied group varied with sexual orientation as the surveyed individuals were not asked to determine it. Some previous studies have, however, shown that homo- and bisexual subjects may be disproportionately high consumers of pornography [80]. Finally, the associations between the religiousness of the surveyed individuals and pornography use was not evaluated. As shown, religion may be an additional factor causing distress to pornography users [81]. 

## 5. Conclusions

The present study reports the high frequency of pornography use among Polish students, and reports that its patterns of consumption may be similar in females and males, although the former tended to be more often embarrassed by such activity. The early age of first exposure (≤12 years) was found to be significantly associated with various self-perceived negative outcomes of pornography use manifesting themselves during university students age (18–26 years). This finding supports the notion that enforcement of age-verified restriction in order to protect child and adolescent individuals from early exposure may be beneficial although one should note that this study has a cross-sectional nature and does not prove causation. 

## Figures and Tables

**Table 1:** Demographic characteristic of the studied group of university students (*n* = 6463).

Parameter	Male (*n* = 2633)	Female (*n* = 3830)
	Mean ± SD (min–max)	Mean ± SD (min–max)
**Age** (years)	22.1 ± 1.7 (18–26)	22.1 ± 1.7 (18–26)
**Body Mass Index** (kg/m^2^)	23.5 ± 3.5 (11.2–56.1)	22.0 ± 3.7 (9.4–70.6)
	n (%)	n (%)
Underweight (<18.5) Normal weight (18.5–24.9) Overweight (25.0–29.9) Class I obesity (30.0–34.9) Class II obesity (35.0–39.9) Class II obesity (>40.0)	93 (3.5) 1791 (68.0) 635 (24.1) 96 (3.6) 15 (0.6) 3 (0.1)	495 (12.9) 2680 (70.0) 524 (13.7) 97 (2.5) 23 (0.6) 11 (0.3)
**Romantic relationship status**		
Single / In a relationship	1499 (56.9)/1134 (43.1)	1402 (36.6)/2428 (63.4)
	n/n (%/%)	n/n (%/%)
**Personality traits**		
Optimistic/Pessimistic Extroversive/Introversive Confident/Shy Curious/Uninquisitive Sensitive/Insensitive Happy/Sad Calm/Aggressive Trusting/Conscious Sociable/Unsociable	1681/952 (63.8/36.2) 2633/1742 (66.2/33.8) 1430/1203 (54.3/45.7) 2322/311 (88.2/11.8) 2240/393 (85.1/14.9) 1957/676 (74.3/25.7) 2279/354 (86.6/13.4) 1525/1108 (57.9/42.1) 1815/818 (68.9/31.1)	2289/1541 (59.8/40.2) 1545/2285 (40.3/59.7) 1988/1842 (51.9/48.1) 3489/341 (91.1/8.9) 3503/327 (91.5/8.5) 2908/922 (75.9/24.1) 2990/840 (78.1/21.9) 2120/1710 (55.4/44.6) 2789/1038 (72.9/27.1)

SD–standard deviation.

**Table 2:** The self-perceived effects reported by surveyed individuals during attempts to cease pornography use (*n* = 2169).

Effects	Occurrence (%)	Severity of Effect (%)			
		Minorly Disruptive	Moderately Disruptive	Severely Disruptive	Very Severely Disruptive
Insomnia	11.7	49.8	30.8	13.0	6.3
Irritability	26.4	54.6	27.9	11.9	5.6
Hands trembling	5.1	49.5	31.5	13.5	5.4
Aggression	14.0	59.2	22.7	11.2	6.9
Anxiety	9.0	47.2	27.2	17.9	7.7
Libido decrease	17.3	50.9	30.7	10.9	7.5
Depression	13.1	43.5	28.3	16.6	11.7
Erotic dreams	53.5	32.9	42.2	17.0	7.8
Attention disturbance	26.0	48.0	30.0	13.7	8.3
Sense of loneliness	22.2	41.7	26.0	17.9	14.4

**Table 3:** The odds ratio (95% confidence interval) for different effects of pornography use in association with age of first exposure in female (*n* = 3004) and male (*n* = 2079).

Effect	Sex	Quartiles of Age of First Exposure to Pornography (Years)			
		Q4 (>16)	Q3 (15–16)	Q2 (13–14)	Q1 (≤12)
Need for longer stimulation to reach orgasm	Female	1.0 (reference)	1.16 (0.81–1.66) *p* > 0.05	1.79 (1.30–2.5) *p* < 0.001	1.62 (1.09–2.41) *p* < 0.05
	Male		1.06 (0.68–1.66) *p* > 0.05	1.58 (1.06–2.36) *p* < 0.05	2.41 (1.52–3.80) *p* < 0.001
Need for more sexual stimuli to reach orgasm	Female		1.56 (1.13–2.17) *p* < 0.01	2.37 (1.76–3.20) *p* < 0.001	2.64 (1.86–3.74) *p* < 0.001
	Male		1.06 (0.74–1.53) *p* > 0.05	1.51 (1.01–2.09) *p* < 0.05	1.94 (1.31–2.88) *p* < 0.001
Decrease in sexual satisfaction	Female		0.92 (0.70–1.20) *p* > 0.05	1.73 (1.36–2.20) *p* < 0.001	2.21 (1.65–2.94) *p* < 0.001
	Male		1.32 (0.94–1.83) *p* > 0.05	1.90 (1.40–2.57) *p* < 0.001	2.40 (1.67–3.46) *p* < 0.001
Decrease in romantic relationship quality	Female		1.01 (0.70–1.46) *p* < 0.05	1.87 (1.36–2.57) *p* < 0.001	2.16 (1.49–3.14) *p* < 0.001
	Male		1.15 (0.76–1.75) *p* > 0.05	1.91 (1.31–2.77) *p* < 0.001	1.87 (1.20–2.93) *p* < 0.01
Neglecting basic needs	Female		1.15 (0.81–1.64) *p* > 0.05	2.04 (1.50–2.79) *p* < 0.001	2.30 (1.61–3.30) *p* < 0.001
	Male		1.66 (1.07–2.57) *p* < 0,05	2.52 (1.69–3.76) *p* < 0.001	3.20 (2.04–5.04) *p* < 0.001
Neglecting everyday duties	Female		1.30 (0.94–1.81) *p* > 0.05	2.75 (2.06–3.67) *p* < 0.001	3.07 (2.20–4.28) *p* < 0.001
	Male		2.16 (1.43–3.28) *p* < 0.001	3.56 (2.43–5.21) *p* < 0.001	3.82 (2.48–5.90) *p* < 0.001
Self-perceived pornography addiction	Female		1.95 (1.32–2.89) *p* < 0.01	3.73 (2.61–5.31) *p* < 0.001	4.23 (2.85–6.28) *p* < 0.001
	Male		3.46 (2.01–5.99) *p* < 0.001	5.94 (3.55–9.94) *p* < 0.001	7.25 (4.16–12.63) *p* < 0.001
